# PRISM (Program of Resources, Information and Support for Mothers): a community-randomised trial to reduce depression and improve women's physical health six months after birth [ISRCTN03464021]

**DOI:** 10.1186/1471-2458-6-37

**Published:** 2006-02-17

**Authors:** Judith Lumley, Lyndsey Watson, Rhonda Small, Stephanie Brown, Creina Mitchell, Jane Gunn

**Affiliations:** 1 Mother and Child Health Research, La Trobe University, 251 Faraday St, Carlton, Victoria 3053, Australia; 2 School of Nursing and Midwifery, La Trobe University, Bundoora, Victoria 3086, Australia; 3 Department of General Practice, University of Melbourne,200 Berkeley StMelbourne, Victoria 3053, Australia

## Abstract

**Background:**

In the year after birth one in six women has a depressive illness, 94% experience at least one major health problem (e.g. back pain, perineal pain, mastitis, urinary or faecal incontinence), 26% experience sexual problems and almost 20% have relationship problems with partners. Women with depression report less practical and emotional support from partners, less social support, more negative life events, and poorer physical health and see factors contributing to depression as lack of support, isolation, exhaustion and physical health problems. Fewer than one in three seek help in primary care despite frequent health care contacts.

**Methods:**

Primary care and community-based strategies embedded in existing services were implemented in a cluster-randomised trial involving 16 rural and metropolitan communities, pair-matched, within the State of Victoria, Australia. Intervention areas were also provided with a community development officer for two years. The primary aim was to reduce the relative risk of depression by 20% in mothers six months after birth and to improve their physical health. Primary outcomes were obtained by postal questionnaires. The analysis was by intention-to-treat, unmatched, adjusting for the correlated nature of the data.

**Results:**

6,248 of 10,144 women (61.6%) in the intervention arm and 5057/ 8,411 (60.1%) in the comparison arm responded at six months, and there was no imbalance in major covariates between the two arms. Women's mental health scores were not significantly different in the intervention arm and the comparison arm (MCS mean score 45.98 and 46.30, mean EPDS score 6.91 and 6.82, EPDS ≥ 13 ('probable depression') 15.7% vs. 14.9%, Odds ratio_adj _1.06 (95%CI 0.91–1.24). Women's physical health scores were not significantly different in intervention and comparison arms (PCS mean scores 52.86 and 52.88).

**Conclusion:**

The combined community and primary care interventions were not effective in reducing depression, or in improving the physical health of mothers six months after birth.

## Background

Maternal depression is common in the months after birth. We found the point prevalence of probable depression, assessed with the Edinburgh Postnatal Depression Scale (EPDS score ≥ 13) to be between 14 and 17% in three Australian population-based surveys [1-3]. Follow-up of the first survey found that 30% of women who had been depressed at eight to nine months were depressed when their infants were two. Only a third of women who had been depressed had sought help from a health professional. When they did seek help it was from a general practitioner (GP) or a maternal and child health nurse (MCHN) [4]. Only 15% of women with depression had sought help from, or been referred to, a mental health professional [4].

Specific physical health problems such as back or perineal pain, mastitis, haemorrhoids, and urinary incontinence were identified in the second survey as being common as were sexual problems, relationship difficulties and severe fatigue. There were complex associations between physical health problems and depression [5]. Earlier studies in the UK [6,7] found that despite the persistence of symptoms for at least a year only half the affected women sought treatment, with even lower consultation rates for perineal pain (21%), urinary incontinence (27%) [8] or faecal incontinence (14%) [9].

Reluctance to seek help was not because of limited contact with services. MCHNs make a home visit soon after hospital discharge to 94% of mothers, and participation by mothers in visits to MCH centres at two, four and eight weeks, and four to eight months is 87–96%. New mothers' groups run by the MCHN are attended by 60% of first-time mothers [10]. There are lower levels of satisfaction with the service in relation to maternal issues [11]. The mean number of visits to GPs by a mother/baby dyad in the six months following birth is 7.7 [12]. A large survey found that 92% of GPs provided postnatal care, but neither the common physical health problems described above, nor depression, were issues which GPs considered part of the routine six-week check, and both were areas where the GPs rated themselves as not very confident [13]. A more detailed discussion of the evidence is included in the study protocol [14].

In the intervention arm of the *PRISM *trial we aimed to re-focus the existing postnatal health care contacts on maternal physical and mental health (enhanced, evidence-based, primary care), to implement community strategies to increase the availability and accessibility of 'time-out', provide better information about common health problems and local services, with encouragement and incentives to use them (more family and community support for mothers), and to measure the impact of these strategies on maternal health. Theories around social networks and social support were influential in trial development as were the principles of cooperative problem solving, forming coalitions and building capacity for effective local action [15,16]. At the time the trial was developed there were no published trials taking this approach though Regier and colleagues at the National Institute for Mental Health had already argued for the importance of community-based interventions in mental health on very similar grounds: that only a minority seek professional help for mental health problems, when they do they turn to the primary care sector and that even then mental health problems are under-recognised [17].

A detailed discussion of the background and development of PRISM is given on the PRISM website [18-22]. The website is an important resource for viewing the design and implementation of PRISM in context, as it includes examples of the materials developed in the course of the project by the research team and in communities, as well as information on staff training and on monitoring processes.

## Methods

The unit of randomisation was local government authorities (LGA) [23]: because of their responsibility for the Maternal and Child Health Program, their provision of other family/community services, and their responsibility for data on new mothers based on statutory birth notifications from hospitals. LGAs gave informed consent to community participation. The eligible LGAs were all those in Victoria, Australia with 300 to 1,500 births a year, except one metropolitan LGA in the centre of the capital city (Melbourne) and one rural one with shared services and population flow across the State boundary with New South Wales. The 33 eligible LGAs were sent an information package [24] and offered a formal briefing. Twenty-six agreed, 25 followed through and 21 signed a Memorandum of Understanding [25] about randomisation and participation.

After stratification into rural and metropolitan areas all possible pair matches in each stratum were identified, taking into account the size of each area, a rating of current and recent community activity, the annual number of births, non-contiguous boundaries, with one set of eight pairs randomly selected [26]. We could not seek informed consent from individual women prior to the intervention since the interventions were implemented at the community level and there was no way of identifying women in advance of the birth. The Ethics Committees of Monash University (1994) and La Trobe University (1995) approved the project.

### The PRISM intervention program

*PRISM *drew on social ecological theory, with program development around existing high impact 'leverage points' (e.g. MCHNs, GPs and community organisations), encouraging both person-centred and environment-centred change [27].

The objectives in primary care were:

• to increase the recognition of depression in mothers of young children at all contacts;

• to facilitate an active response to the recognition of depression;

• to provide explicit offers of time to talk by both MCHNs and GPs;

• to increase the recognition and treatment of physical and mental health problems which are common after birth;

• to encourage practitioners to think of 'community' solutions to isolation and lack of support.

The strategy was to develop multi-faceted education and training programs for GPs and MCHNs. This involved 10 hours of workshops, simulated patients, two clinical practice audits and evidence-based guidelines (*Guidelines for Assessing Postnatal Problems*) for GPs [28]. A similar education program was provided for MCHNs with 12 hours of training in year one and three hours in year two [29].

The objectives at the community level were:

• to increase the availability and accessibility of support and 'time-out' for recent mothers;

• to provide better information about local services to mothers and families, with encouragement and incentives to use them;

• to increase the 'mother-and baby-friendliness' of local environments (e.g. shopping centre car spaces for parents with prams, improved baby-change facilities);

• to increase inter-organisational collaboration and advocacy for parents and young children.

The key minimum strategies were:

• assessment of the availability and accessibility of facilities and services such as occasional child care, recreational, library, and counselling services, neighborhood houses, local parks), with a focus on the extent to which they were mother-and baby-friendly; an information kit for mothers, distributed to women by MCHNs soon after hospital discharge [30], including:

• a listing of local services for mothers and babies;

• two booklets outlining common physical and emotional health issues for mothers and strategies other women have found helpful, developed by the co-ordinators and piloted with women;

• a booklet for fathers, developed and piloted by a father with fathers;

• a package of free or discounted service vouchers for mothers.

• a range of mother-to mother support strategies based on the principle of non-professional befriending [31].

Local co-ordination was achieved by the establishment of local steering committees with broad membership and a full-time community development officer (CDO) appointed with local selection processes in each intervention community for two years from November 1998. Their duties and responsibilities were to: liaise with local government and non-government agencies and primary care providers; identify local community services, compile information on services for mothers, seek voucher contributions from businesses and local agencies, contribute to the mothers' information kits; facilitate supportive social networks, and provide support to the steering committee in intervention development and implementation, and its subsequent integration into local services and programs. This included working with GP Divisions and with GP liaison officers.

Steering committees and CDOs were able to develop other supportive interventions locally and to decide how to implement them. Extensive communication between the CDOs, discussion on Steering Committees, and articles in the PRISM project newsletters facilitated creative responses to common difficulties and sharing of local strategies [32].

### Health outcomes

Health outcomes were measured by a postal questionnaire mailed six months after birth to women giving birth from 7/02/00 to 5/08/01. Mothers of infants who had died were excluded. Questionnaires were packaged with a covering letter, and a prepaid reply envelope, grouped and mailed to LGAs where a name and address label was added from their MCH program data system. Reminder cards were sent two and four weeks later. Questionnaires were returned direct to the research team to ensure anonymity and confidentiality. The primary outcome measures used to assess women's health and well-being at six months were the EPDS (a 10-item scale developed for use in the postnatal period, in which a score ≥ 13 identifies probable depression [33], and the physical and mental component scores (PCS and MCS) of the Short Form 36 (SF-36), a widely used general health status measure [34]. The PCS and MCS were calculated using norms from the 1995 Australian National Health Survey [35] using appropriate female age-group subscale means.

Other questions included women's views of the practical and emotional support they had received, social contacts, making new friends, 'time out', mother and baby friendliness of local settings, receipt and use of the mothers' information kit and vouchers. We asked about the extent to which their own GP and MCHN were supportive and understanding, and about their use of other health services.

### Sample size and power

The sample size to detect a relative risk reduction in probable depression (EPDS ≥ 13) of just under 20%, (an absolute difference of 3%), given the depression prevalence of 14 to 17% and individual randomisation, with α = 0.05 (2-sided), β = 0.20, would be 2337 in each arm. To allow for the pair-matched cluster randomisation design it was estimated that with eight pairs, and an average cluster size of 800, a matching correlation of at least 0.3 would be required; an inflation factor of approximately 2.5 [36]. This sample size would be able to detect two point differences of clinical importance in the summary mental and physical scores of the SF-36. Adjustment for a likely adjusted response fraction of 67%, based on earlier surveys of recent mothers [1-3], increased the required sample size to 9,600 per arm. Routine monitoring of responses during the trial showed a lower response fraction than predicted. For this reason the data collection was extended to 18 months of births with the support of all 16 participating LGAs [14].

### Analysis

Two methods of analysis have been suggested for cluster-randomised trials with categorical outcomes: the logistic-normal characterised as 'cluster-specific' and the 'population-averaged' model using the generalised estimating equations (GEE) extension of logistic regression. The PRISM trial was analysed using the former method. Given the large size of each cluster (adjusted average 695) and the consequently small intra-class correlation (approximately 0.0012) the two methods would provide approximately equal solutions. Pair-matches were broken in the model to provide more power [37]. Linear regression was used for the analysis of other health outcomes. The analysis was carried out with Stata, version 8.

## Results

**Figure 1:**
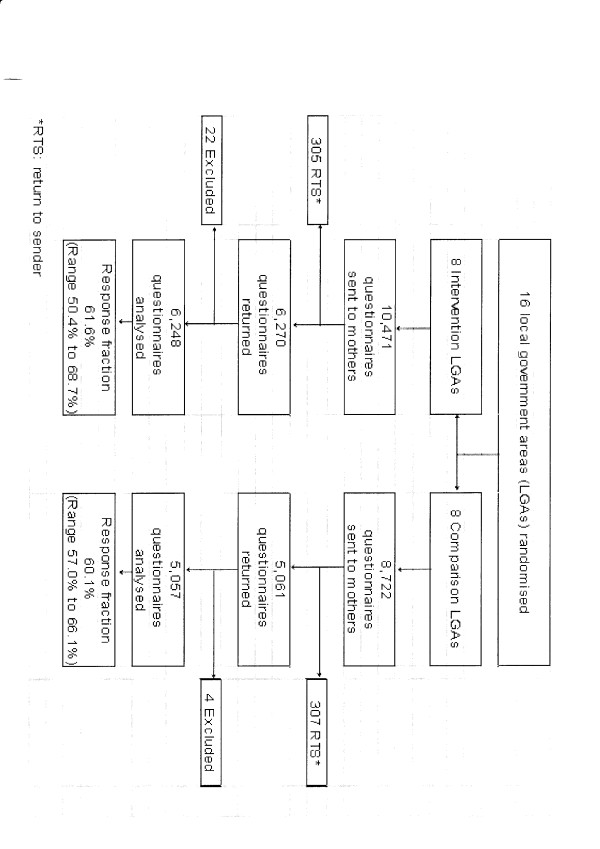


Figure 1 shows the participant flow diagram [38]. No clusters were lost from the study. The adjusted response fractions were 6,248/10,144 (61.6%, range 50.4% to 68.7%) from intervention (I) communities and 5,057/8,411 (60.1%, range 57.0% to 66.1%) from comparison (C) communities. Two women were inadvertently included who gave birth outside the birth-date range but within a week. Characteristics of the clusters in terms of remoteness, size (area) of the LGA, number of births in the study period, family income, and proportions with post-secondary qualifications [39-41] are listed in Table 1. This Table shows the comparability of the intervention and comparison clusters.

Table 2 summarises the social and reproductive characteristics of the survey participants by group showing similar proportions in social and perinatal characteristics. There were no differences in infant sex with 51.7% male, multiple births 1.5% (90 twin, 4 triplet (I), 72 twin, 1 triplet, 1 quadruplet (C)), identification as Indigenous (23 (I), 29 (C)), or giving birth outside a hospital (45 (I), 20 (C)). Data on all women giving birth in the 16 communities during the study period, obtained from the Victorian Perinatal Data Collection Unit (VPDCU) are also shown in Table 2. Survey respondents included fewer women who were under 20 or 20–29, Indigenous, without a partner, of non-English-speaking background, or without private health insurance.

**Figure 2:**
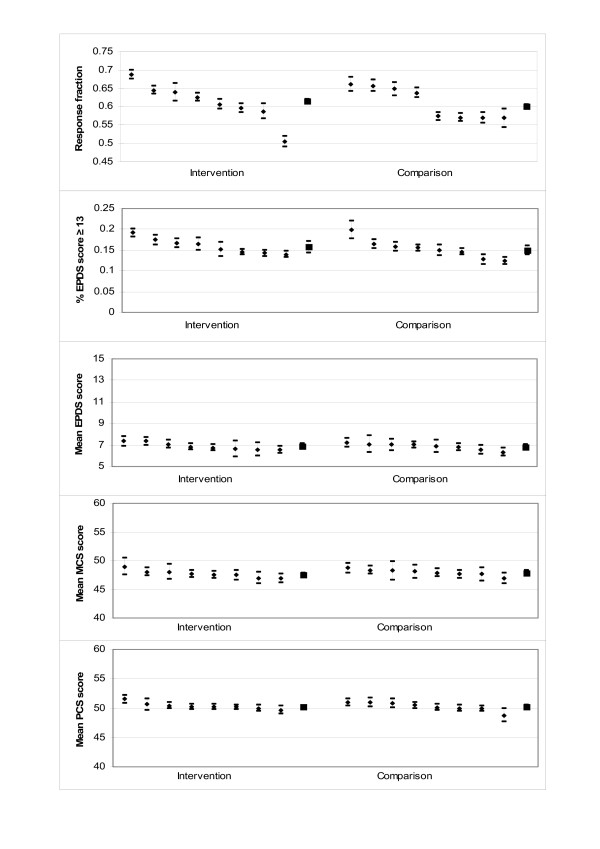


Figure 2 displays response fractions by LGA, for intervention and comparison communities in the top panel. The other panels show the primary outcomes by LGA: the proportion of women with EPDS scores ≥ 13, mean EPDS scores, mean mental health component scores (MCS) and mean physical health component scores (PCS) of the SF-36. There is no evidence of differences between intervention and comparison communities on any outcome.

Table 3 summarises the differences in the major outcome variables across intervention and comparison communities, adjusted for clustering using survey analysis procedures. The proportions of women with probable depression (EPDS ≥ 13) were 15.7% (I) and 14.9% (C), adjusted odds ratio 1.06 (0.91–1.24), and the mean EPDS scores were 6.9 (SE_adj _0.11) and 6.8 (SE_adj _0.11). The mean PCS scores were 50.24 (SE_adj _0.10) and 50.26 (SE_adj _0.16), and the mean MCS scores were 47.58 (SE_adj _0.15) and 47.91 (SE_adj _0.19). Sub-scale scores of the SF-36 are also displayed. Statistical comparisons are shown from univariate analyses as there was no imbalance in key covariates.

### Subgroup analyses

The pre-specified subgroup effects were investigated by examining interactions between the intervention covariates. Where significant interactions occurred stratified analyses were undertaken. Interaction effects between the intervention and pre-specified covariates – rural/metropolitan residence, poverty (family income below and above $30,000 (AUD)), women living with and without a partner, and women's country of birth (to compare women born in Australia, with women born in other English-speaking countries or in non-English-speaking countries) were assessed for all health outcomes. Significant, or near significant, interactions were identified for women without a partner in proportion of women probably depressed, and for women born in non-English-speaking countries, in both EPDS and PCS mean scores. In subsequent stratified analyses of these groups women without a partner were less likely to have probable depression in intervention communities and women of non-English speaking background had lower mean EPDS scores and higher mean PCS scores in intervention communities. The interaction between the three 6-month periods when the birth occurred and the intervention was statistically significant but with the non-significant main effects of time and intervention giving inconsistent effects. [For further information contact the authors].

### Implementation of the intervention

The Mothers' Information Kit was received by 88.2% of women in intervention areas, with only 2.7% of those in comparison areas reporting having received it and 9.3% being unsure. In five LGAs distribution of the kits to mothers was sustained at 90% or more for 18 months. In the other three it fell to 60 to 70% in the last six months. Over 90% of women who received the kit had some positive response to the vouchers, 35% who received the kit had used the vouchers, and 62% rated the local community guide as very or fairly helpful.

The proportions of women reporting receipt of written information were significantly higher in intervention than comparison communities for: information for fathers (60% vs 27%), information on local services for mothers (90% vs 64%), and vouchers or special offers for mothers with a new baby (88% vs 64%) (Table 4). There were no differences between intervention and comparison communities in the proportions of women reporting encouragement to talk about their own health at every visit to the MCHN (36% in both), in those feeling able to talk to their MCHN and finding her very supportive and reassuring (40% and 39%), or in those feeling able to talk to their GP and finding her/him very supportive and reassuring (46% and 43%). Restricting the comparisons to women having more frequent or regular contacts, with MCHNs or GPs, gave the same results. There were no differences in the proportion of women who had made new friends since the birth (53% and 54%), had more social contacts (54% in both), or in women who had time-out, at least once a week, when someone else was caring for the baby (39% and 40%). There was no difference in the proportions describing their local community as very or fairly mother-and-baby friendly (56% and 54%). Despite the marked difference in the proportion of fathers in intervention and comparison communities reported as receiving printed information on 'ways to support you and be involved with the baby' (60% vs 27%), there was no difference between intervention and comparison communities in mothers' rating of partners' practical and emotional support. The mean scores were 6.9 (SE_adj _0.03) (I) and 6.9 (SE_adj _0.07) (C) derived from a set of six questions.

## Discussion

The imbalance of births in intervention and comparison communities in *PRISM *was explained by fewer births in most rural LGAs and rapid population growth in a few metropolitan intervention areas (Victorian Perinatal Data Collection Unit, unpublished data.) The adjusted response fraction was slightly lower in *PRISM *than in our earlier postnatal population surveys [1-3], possibly because we could not afford to send a second copy of the questionnaire, but the differences in social characteristics between all eligible women and survey participants were very similar to prior surveys and the prevalence of probable depression was the same as in earlier surveys [1-3]. As individual consent for participation had not been sought the adjusted response fraction does not demonstrate serious loss to follow-up but rather a relatively high response to receiving a mailed questionnaire 'out of the blue'.

Although the power calculation showed that a particular sample size would be required to identify a statistically and clinically important difference in the primary outcomes between the intervention and comparison groups the finding of no effect of the intervention is strongly based in the similarity of the proportions responding to the outcome questionnaire in the two arms of the trial, and the almost identical primary and secondary outcomes. Thus it is clear that the interventions in this trial did not have an impact on women's mental and physical health at six months after childbirth.

The other universal postnatal intervention trials, those recruiting women across the whole postnatal population, were all designed at the same time, with the exception of Gunn's trial which was a little earlier [42-49]. All used the same mental health outcome measure (see Table 5) and all but one also measured overall health status (mental and physical) with the Short Form 36. The interventions in the six trials were very diverse, although *PRISM *and the trial of MacArthur and colleagues had some components in common. The similar timing of the six trials meant that they were not influenced by the others' findings. The lack of effectiveness of all the interventions implemented in these trials, except that of MacArthur and colleagues, is in contrast to the marked effectiveness of a wide range of postnatal counselling interventions, provided by a variety of practitioners, to women who had been diagnosed as being depressed or probably depressed. The pooled estimate of effect for those interventions is a large reduction in depression: with a relative risk of 0.52 (95% CI 0.40, 0.65) and no significant heterogeneity across the trials [50].

Our hypothesis from the beginning was that the inclusion of physical health as well as mental health and the community-based interventions would make a real contribution beyond the trials focused on individual women. That hypothesis was subsequently borne out by the outcomes of the trial of MacArthur and colleagues which *was *effective in reducing depression [47,48]. Distinguishing features of that trial were its use of existing staff and services to provide redesigned community postnatal care, the integration of their community midwives into primary services and their focus on women's individual physical and psychological health needs. Although there was substantial common ground between MacArthur's trial and *PRISM*, including the finding of no effect on physical health in either trial, there were some differences which may have been important. The lack of integration of MCHNs with other primary care services (general practitioners) in Australia is one and the negative impact of a fee for service system on ready access to a GP in Australia is another. However, the success of MacArthur's trial raises the possibility that *PRISM *could have been more effective, and we consider below a number of possible explanations for why it was not.

The impact of education and training on primary caregivers in *PRISM*, assessed in terms of women's ratings of their care, was much less than we had hoped for. There was a real but small impact on GPs taking part in the education program [28] but these were a small proportion of all GPs in participating communities, and academic detailing was limited. We saw the role of MCHNs in *PRISM *as pivotal but recognised that the education and training in *PRISM *involved a role shift from a focus on action around the health and well-being of babies, child health surveillance, immunisation and child protection, to a much more open-ended role involving 'active listening' to mothers, enhanced communication skills and much less certainty about what should be done [51].

The CDOs had a five-day residential training program at the start of employment, eight all day meetings as a group with the research team, and three all day meetings with a range of participating community representatives in Melbourne, as well as having frequent email and telephone contact with each other and with the PRISM co-ordinators [22]. However, their employment was for only two years which may not have been long enough, especially given the changes to local government (described below) which militated against community building.

The negligible impact of the whole intervention on women's partners was disappointing, and could have been a limiting factor in the effectiveness of the intervention in improving women's health outcomes.

One explanation for the lack of effect of the intervention might have been that the elements of *PRISM *or other major alternative maternal health programs were implemented in comparison communities. Local government changes made that unlikely but we assessed the evidence in 2001–2 through 'unobtrusive monitoring' [52] of, policies, programs and funding at local, state, and commonwealth government levels [53], and an audit of GP Divisional projects, strategic plans and business plans. We also analysed systematic samples of local newspaper coverage of mothers and maternal health [54] and surveyed the MCH team leaders in each comparison community to ask about specific local initiatives, finding almost none.

In the five years this trial was being planned (1993–1997) there were marked changes to local government implemented by the State government, including the dismissal of elected local councillors, appointment of commissioners, and the amalgamation of local councils from 210 to 78 [55]. Service-contracting became a prominent feature of councils' operation for the first time, with a requirement that at least half of all municipal services be put out to compulsory competitive tendering – including, in most municipalities, the MCH Program [56]. 'In-house' business units, comprising staff previously employed to provide the service directly, won some of the contracts for health and family support services. Some were won by community-based agencies, e.g. community health centres, some by hospitals, and some by private companies. Although the straitened funding co-incident with the reforms made the 50% chance of being provided with resource kits for mothers, professional development for MCHNs and a CDO for two years very attractive, the enforced competition was not the ideal context for a community intervention [57].

## Conclusion

Given the study size, the comparability of the two arms, the evidence of implementation, and the almost identical health outcomes on all measures, it is most unlikely that this complex multi-faceted intervention improves maternal physical or psychological health.

## Competing interests

The author(s) declare that they have no competing interests.

## Authors' contributions

JL, RS, LW and SB conceived the trial design and the intervention and wrote the first successful grant application.

RS and SB were jointly responsible for the co-ordination and implementation of *PRISM*. They developed the training program for maternal and child nurses, with input from JG.

JG developed the training program for GPs within a separate but linked project and wrote the *Guidelines for Assessing Postnatal Problems (GAPP)*.

JL, RS, SB, CM, JG and LW were involved in the development of the questionnaire sent to women six months after birth.

CM developed the data collection and monitoring systems with participating communities, and was responsible for piloting, data management, coding, and cleaning.

LW provided statistical expertise, oversight of the randomisation, and carried out the analysis with CM.

All the authors were members of the research team which met regularly through the project. All contributed to the selection of variables for analysis and all have commented on the drafts of the paper.

JL was responsible for the overall direction of the project and is the guarantor for the paper.

## Pre-publication history

The pre-publication history for this paper can be accessed here:



## Figures and Tables

**Table 1:** Characteristics of the clusters in intervention and comparison communities

		Intervention	Comparison
Area km^2^	< 50	1	2
	50–99	1	0
	100–999	2	2
	1000–4999	2	3
	≥ 5000	2	1
			
Accessibility & remoteness	Highly accessible	6	7
	Accessible	2	0
	Accessible/mod. accessible	0	1
	Remote	0	0
			
Proportion of households with a weekly income ≥ $1000 [AUD]	<15%	1	2
	15–19%	4	2
	20–24%	0	1
	25–29%	2	1
	≥ 30%	1	2
			
Proportion of households where no person has a post-secondary qualification	<45%	0	1
	45–49%	1	0
	50–54%	0	0
	55–59%	2	1
	60–64%	3	3
	≥ 65%0	2	3
			
Number of births in study period	>1000	2	1
	750–999	2	2
	500–749	2	2
	<500	2	3
			
Adjusted response fraction, health outcomes questionnaire	>65%	1	2
	60–64%	4	2
	55–59%	2	4
	<55%	1	0

**Table 2:** Characteristics of women and births in intervention and comparison communities, compared with all women and births in PRISM areas

	PRISM	(I) N = 6248	PRISM	(C) N = 5057	All births N = 20,333	
	N	*%*	N	*%*	N	*%*
						

*Place of residence*						
Rural	4035	*64.6*	3475	*68.7*	13352	*65.7*
Metropolitan	2213	*35.4*	1582	*31.3*	6981	*34.3*
						
*Maternal age (years)*						
<20	100	*1.6*	56	*1.1*	734	*3.6*
20-24	574	*9.2*	383	*7.6*	2593	*12.8*
25–29	1735	*27.8*	1332	*26.3*	282	*30.9*
30–34	2371	*37.9*	1990	*39.4*	966	*34.3*
>34	1346	*21.5*	1205	*23.8*	3757	*18.5*
Missing	122	*2.0*	91	*1.8*	1	*0.0*
						
*Highest education level*						
Degree	1602	*25.6*	1508	*29 8*	NA†	NA
Diploma/Apprenticeship	1864	*29.8*	1392	*27.5*		
Completed secondary	1203	*19.3*	922	*18 3*		
Did not complete	1538	*24.6*	1199	*23.7*		
Missing	41	*0.7*	36	*0.7*		
						
*Marital status*						
Married	4903	*78.5*	4129	*81.6*	4690	*72.2*
Living with partner	956	*15.3*	677	*13.4*	2999	*14.7*
Single	253	*4.0*	154	*3.0*	2377	*11.7*
Separated/Widowed/Divorced	120	*1.9*	74	*1.5*	249	*1.2*
Missing	16	*0.3*	23	*0.5*	18	*0.1*
						
*Family income before tax*						
≤ $30,000 [AUD]	1303	*20.9*	879	*17.4*	NA	NA
$30,000 to < $70,000	3066	*49.1*	2394	*47.3*		
>$70,000	1566	*25.1*	1498	*29.6*		
Missing	313	*5.0*	286	*5.7*		
						
*Country of birth/language*						
Australia	5481	*87.7*	4315	*85.3*	16999	*83.6*
Other English-speaking	485	*7.8*	338	*6.7*	1373	*6.8*
Other non-English speaking	265	*4.2*	379	*7.5*	1945	*9.6*
Missing	17	*0.3*	25	*0.5*	26	*0.1*
						
*Gestation length in weeks*						
20–27	18	*0.3*	15	*0.3*	62	*0.6*
28–31	37	*0.6*	21	*0.4*	131	*0.6*
32–36	349	*5.6*	294	*5.8*	1110	*5.5*
37–41	5600	*89.6*	4524	*89.5*	8731	*92.1*
>41	130	*2.1*	81	*1.6*	295	*1.5*
Missing	114	*1.8*	122	*2.4*	4	*0.0*
						
*Parity*						
Primiparous	2704	*43.3*	2238	*44.3*	8274	*40.7*
Multiparous	3533	*56.5*	2803	*55.4*	12059	*59.3*
Missing	11	*0.2*	16	*0.3*	0	*0.0*
						
*Private health insurance‡*						
Medicare only	3957	*63.3*	3054	*60.4*	14004	*68.9*
Private cover	2254	*36.1*	1960	*38.8*	6329	*31.1*
Missing	37	*0.6*	43	*0.9*	0	*0.0*
						
*Postnatal length of stay*						
<24 hours	173	*2.8*	142	*2.8*	NA	NA
1–2 days	960	*15.4*	702	*13.9*		
3–4 days	2326	*37.2*	1845	*36.5*		
>4 days	2771	*44.4*	2355	*46.6*		
Not available	17	*0.3*	5	*0.1*		
Missing	1	*0.0*	8	*0.2*		
						
*Type of birth*						
Spontaneous vaginal	3830	*61.3*	2972	*58.8*	12740	*61.6*
Forceps/vacuum	937	*15.0*	814	*16.1*	2799	*13.8*
Elective Caesarean	821	*13.1*	706	*14.0*	2745	*13.5*
Emergency Caesarean	634	*10.1*	540	*10.7*	2381	*11.7*
Missing	26	*0.0*	25	*0.5*	1	*0.0*
						
*Infant birth-weight*						
<2500 g	343	*5.4*	215	*4.3*	1274	*6.2*
2500–3999 g	4909	*77.4*	3981	*78.7*	16803	*81.3*
≥ 4000 g	890	*14.0*	664	*13.1*	2588	*12.5*
Missing	204	*3.2*	197	*3.9*	1	*0.0*

I Intervention C Comparison

Data on all births was provided by the Victorian Perinatal Data Collection Unit

† Not available in the perinatal data collection

‡ PRISM data is self-report of health insurance status, PDCU data is accommodation status in hospital

**Table 3:** EPDS 'probable depression' and mean scores, SF-36 mean scores and subscales, six months after birth

	Intervention (n = 6248)			Comparison (n = 5057)			Statistical tests			
	n	mean or %	SE_adj_	n	mean or %	SE_adj_	p-value	OR	SE_adj _(95% CI)	DEFF

**EPDS ≥ 13**	6221	15.72%	0.75%	5027	14.94%	0.55%	0.41	1.06	0.08 (0.91 to1.24)	1.81
								Difference		
										
**EPDS, mean score**	6163	6.91	0.11	4969	6.83	0.11	0.61	0.08	0.09 (-0.25 to 0.40)	2.28
										
**SF-36 PCS†**	5917	50.24	0.10	4761	50.26	0.16	0.91	-0.02	0.19 (-0.43 to 0.39)	1.71
										
**MCS‡**	5917	47.58	0.15	4761	47.91	0.19	0.20	-0.32	0.24 (-0.83 to 0.18)	1.27
										
**SF-36 Subscales§**										
PF	6163	89.72	0.24	4979	89.41	0.46				
RP	6090	81.38	0.45	4923	81.70	0.51				
BP	6233	78.85	0.28	5031	79.65	0.19				
GH	6177	74.65	0.46	4971	75.65	0.62				
Vitality	6219	51.29	0.31	5025	52.16	0.39				
SF	6238	81.93	0.25	5039	82.40	0.30				
RE	6082	80.99	0.57	4895	81.70	0.42				
MH	6219	72.57	0.30	5024	72.85	0.33				
Health transition	6240	2.94	0.02	5045	2.97	0.01				

† Physical health component score, adjusted for age/sex distribution of PRISM population, factor loadings and standard deviation using Australian National Health Survey values

‡ Mental health component score, adjusted as for PCS

§ PF physical functioning, RP role functioning (physical), BP bodily pain, GH general health, SF social functioning, RE role functioning (emotional), MH mental health.

**Table 4:** Women's reports of aspects of the intervention, primary caregiver support, community support, and partner support, in intervention and comparison communities

	Intervention		Comparison		OR (95% CI)	p-value
	N	%	N	%		

Received printed information about own health after birth of the baby	5080	81.3	3769	74.5	1.50 (0.94–2.38)	0.08
Printed information about own health rated very or fairly helpful †	3285	64.7	2556	67.8		
Received printed information for fathers on ways to support you and be involved with baby	3761	60.2	1380	27.3	4.24 (3.63–4.95)	<0.001
Received printed information on local services for mothers	5648	90.4	3246	64.2	5.36 (3.53–8.20)	<0.001
Printed information on local services rated very or fairly helpful †	3013	53.3	1728	53.2		
Received vouchers or special offers for mothers with a new baby	5473	87.6	3032	60.0	4.81 (2.84–8.14)	<0.001
Encouraged to talk about own health by MCHN at every visit	2237	35.8	1807	35.7		
MCHN very supportive and understanding	2486	40.0	1961	39.0		
GP very supportive and understanding	2846	39.0	2180	43.1		
Made new friends since the baby	3329	53.3	2734	54.1		
More social contacts in the local community since the birth	3349	54.1	2738	54.1		
'Time-out' at least once a week	2439	39.0	1995	39.5		
Local community:						
Very mother & baby-friendly	1024	16.4	833	16.5		
Fairly	2488	39.8	1874	37.1		
Mixed or not helpful	2638	42.2	2276	45.0		
	N	Mean (SE_adj_)	N	Mean (SE_adj_)		
Partner support score‡ [based on six questions, possible scores 0 to 10]	5859	6.88 (0.03)	4806	6.85 (0.07)		
Missing	128		97			

† Analysis restricted to those who reported having received the information

‡ Analysis restricted to women married or living with a partner

**Table 5:** Mental health outcomes in universal postnatal intervention trials

Author and year	Outcome measure	Timing of outcome assessment	Prevalence of probable depression I vs C (%)	RR [95% CI] of depression OR [95% CI] of depression *EPDS mean score*
Gunn 1998 [39]	EPDS ≥ 13 EPDS mean score	3 months 6 months 3 months 6 months	16.8 vs 13.6 11.6 vs 12.8	RR 1.24 [0.81 to 1.90] RR 0.91 [0.56 to 1.48] *7.38 vs 7.48* * 5.87 vs 6.08*
Morell 2000 [40,41]	EPDS ≥ 12 EPDS mean score	6 weeks 6 months 6 weeks 6 months	17.8 vs 18.0 18.9 vs 21.6	RR 0.98 [0.69 to 1.41] RR 0.89 [0.62 to 1.27] *7.4 vs 6.7 * *6.6 vs 6.7*
MacArthur 2002 [44, 45]	EPDS ≥ 13, multi-level modelling EPDS mean of cluster means	4 months 4 months	14.4 vs 21.3	OR_adj _0.57 [0.43 to 0.76] *6.40 vs 8.06*
Reid 2002 [42, 43]	EPDS ≥ 12 EPDS mean score	3 months 6 months 3 months 6 months	14.6 vs 11.7 14.8 vs 9.6	RR_w _1.21 [0.79 to 1.85] ¶ RR_w _1.55 [0.95 to 2.52] ¶ *C P G PG* 5.9 vs 5.6 vs 6.1 vs 6.1 *5.0 vs 5.7 vs 5.4 vs 5.3*
Priest 2003 [46]	Depression by DSM-IV criteria	Depression diagnosis during 1^st ^postnatal year	17.8 vs 18.2	RR 0.98 [0.80 to1.02]
PRISM 2005	EPDS ≥ 13 EPDS mean score	6 months 6 months	15.7 vs 14.9	OR _adj _1.06 [0.91 to 1,24] *6.8 vs 6.9*

¶ Probable depression outcomes for the intervention subgroups (information pack ((P), invitation to group (G) or both (PG)) were pooled and compared with standard care (no pack, no group); RR_w _Mantel-Haenszel weighted relative risk
